# Improvement of radiographer commenting accuracy of the appendicular skeleton following a short course in plain radiography image interpretation: A pilot study

**DOI:** 10.1002/jmrs.306

**Published:** 2018-10-09

**Authors:** Imelda Williams, Marilyn Baird, Beverley Pearce, Michal Schneider

**Affiliations:** ^1^ Department of Medical Imaging and Radiation Sciences Monash University Clayton Victoria Australia; ^2^ Peninsula Health: Frankston Hospital Frankston Victoria Australia

**Keywords:** Boundaries/roles, education, general radiography, image interpretation, radiography

## Abstract

**Introduction:**

Radiographers are at times required to provide preliminary information on plain radiography when significant findings are identified. The aim of the study was to evaluate the effectiveness of two short training modules to improve the accuracy of image interpretation of the appendicular skeleton amongst a group of radiographers.

**Methods:**

Eight radiographers volunteered to participate in the study. All undertook a pre‐test and, following delivery of course materials, an immediate post‐test for two consecutive modules. A retention test was undertaken 6 months later. Sensitivity (Sn), specificity (Sp) and accuracy (Acc) scores were evaluated against the “Gold Standard” radiologists’ reports. Paired‐samples *t*‐tests were carried out to compare image interpretation scores between the start of module one to the end of module two, and between the end of module 2 and 6 months later. Summary receiver operating characteristics (SROC) scores on each of the participants’ module two post‐test study results were undertaken.

**Results:**

Significant improvements in scores were achieved between the mean (SD) scores of module 1 pre‐test (77.5 (±3.9)) and the module 2 post‐test (83.6 (±3.2) (*P* =0.022)). Sn, Sp and Acc scores increased from the start of module 1 pre‐test to the end of module 2 post‐test (Sn: 82.28–86.25%; Sp: 75.29–84.66%; Acc: 81.68–85.97%). The retention test revealed a non‐significant reduction in mean scores (80.0 (±5.1)) when compared to post‐test module 2 (83.6 (±3.2) (*P* =0.184)). SROC revealed an area under the curve of 0.90.

**Conclusion:**

Participants achieved significant improvements in commenting accuracy on plain radiography of the appendicular skeleton after completion of the two modules. However, continuous application and ongoing professional development is essential in order to maintain and develop the skills acquired.

## Introduction

The Medical Radiation Practice Board of Australia (MRPBA) in the published document titled “Professional capabilities for medical radiation practice”^1^ has an expectation under Domain 5 that radiographers should be able to convey knowledge about significant findings on plain radiography to referring practitioners in either verbal or written form. Identifying significant findings requires radiographers to apply their knowledge of radiographic anatomy and abnormal imaging appearances and relating these appearances to the patient's clinical history.

Whilst university programs prepare their students to meet this expectation, the Chair of the MRPBA stated in the December 2017 newsletter,^2^ that in other cases, a training program may have to be developed that require agreement with the employer in order to meet the minimum requirements in that additional area of practice. A 2014 study^3^ suggested that, as an alternative to formal post‐graduate programs, targeted image interpretation training be delivered as an intensive (two‐consecutive 6‐h days) or non‐intensive (90‐min weekly tutorials conducted over 2 months) format. As the provision of radiographic comments on plain radiography has not been routine practice, Peninsula Health, Frankston Hospital contracted the Department of Medical Imaging and Radiation Sciences at Monash University to deliver a non‐award ‘Short Course in Advanced Radiographic Clinical Skills’ as a pilot project for radiographers. The short course aimed to extend the knowledge of participants in the field of emergency image interpretation, focusing on plain radiography representation of traumatic and common pathologies affecting the appendicular skeleton.

### Historical perspective

The radiologist Dr Swinburne suggested in 1971 that radiographers had the potential to comment on radiographic images.^4^ This suggestion was reinforced in 1980 when De Lacey and others recognised that errors in emergency departments could be reduced by using radiographers to flag abnormalities.^5^, ^6^, ^7^ It was during the early 1980s that an abnormality detection scheme ‘Red Dotting’ was introduced in the United Kingdom (UK).^8^, ^9^ A Queensland state ministerial taskforce^10^ argued that improvements to patient‐centred care, as well as service effectiveness and efficiency could be achieved by implementation of a full scope of practice for Allied Health professionals. Numerous studies have reported that tailored training in pattern recognition could improve radiographer's ability to identify fractures and dislocations and provide descriptive comments.^11^, ^12^, ^13^ Radiographers have an ethical and professional responsibility to the Australian public to provide an opinion on images produced using their knowledge and experience as expected by the MRPBA's professional capabilities document.^1^ Image interpretation is integrated in Australian accredited radiographer undergraduate and graduate entry programs, preparing graduates to provide a valuable skill that can facilitate patient management at the point‐of‐care.^1^ However, new graduates will still need continuous professional education in radiographer abnormality detection schemes to further develop their image interpretation skills.^14^, ^15^ Short course training for radiographers has not been evaluated in Australia to date. Hence, this pilot study was carried out in order to evaluate the effectiveness of two short training modules to improve the accuracy of image interpretation of the appendicular skeleton amongst a group of radiographers. A straw poll was administered amongst medical imaging practices to gain an opinion on whether radiographer commenting was regularly performed.

## Methodology

Ethics approval was obtained from the Monash University Human Research Ethic committee (MUHREC Project Number: CF16/2230 – 2016001102) and Peninsula Health Ethics Committee. Purposive sampling recruited eight radiographers from Peninsula Health, Frankston hospital, Medical Imaging Department in the State of Victoria who volunteered to participate in this study. Interested radiographers had to submit a written statement of intent. An independent panel selected successful participants. Radiographers had to demonstrate genuine interest in image interpretation as well as availability to complete both the education modules within the required timelines. All participants (*n *= 8) consented to participate in the pilot project. The participants enrolled into the following modules:

☐ Module 1: Appendicular skeleton of the shoulder girdle and upper limbs; and
☐ Module 2: Appendicular skeleton of the pelvic girdle and lower limbs.

The short course was hosted via the Monash University, Faculty of Medicine, Nursing and Health Sciences “Health Professional Education Online” learning site. Each module had a study period of 16 weeks. Formative (60 cases) and summative test banks (125 cases) included plain radiography cases to assess a variety of normal and traumatic conditions as well as common pathologies as displayed in Table 1. The prevalence of normal to abnormal cases was 25% normal (including normal variants) and 75% abnormal cases. Abnormal cases also included images with more than one radiographic injury to test participants’ ability to implement a search strategy. Test bank images were complemented with the use of a dedicated image manipulation software program, iQ‐View (Wodonga, Australia). Participants were required to provide comments on a dedicated opinion form based on the ‘ABCS Search Strategy’.^16^ Prior to the start of each module, participants undertook a randomly selected 25 case pre‐test which included adult and paediatric normal and traumatic pathology to examine their base knowledge of image interpretation and commenting skills specific to each of the education modules. The subsequent course material was delivered online which included video‐recorded PPT presentations, access to an online ‘Image Interpretation Workbook’ and a hard‐copy textbook.^16^ Following delivery of the dedicated material, participants completed a formative test comprising 10 cases reflective of the specific body region under investigation. The primary researcher provided immediate video‐recorded feedback on each of the formative practice banks upon completion of each formative test. Each module concluded by participants undertaking a second randomly selected 25 case post‐test to examine whether participants’ image interpretation scores improved across the delivery of the two modules. Participants also provided a written comment on each of the cases. The comment marking criteria was scored against the ‘Gold Standard’ radiologists reports. Participants’ comments needed to answer the clinical question for each of the cases. Unrelated incidental radiological findings that did not influence the patient management were excluded from the marking criteria. Six months after completing the post‐test for module two, participants undertook a randomly selected 25 case retention test to examine their long‐term ability to identify and comment on normal and abnormal radiographic appearances of the appendicular skeleton. The retention test included upper and lower limb cases with a 1:4 normal to abnormal ratio. Responses were classified as true positive (TP), true negative (TN), false positive (FP) or false negative (FN) against the ‘Gold Standard’ radiologists’ reports, using partial marks in case of multiple abnormalities present. Aggregated sensitivity (Sn), specificity (Sp) and accuracy (Acc) rates as well as likelihood ratios, negative predictive value (NPV) and positive predictive value (PPV) were analysed. SCOR scores of the module two post‐test revealed the individual participants’ sensitivity and specificity achieved immediately after the delivery of the two modules.^17^ In addition, a Qualtrics electronic single‐question survey was distributed to all registered Victorian public and private Medical Imaging practices (*n* = 129) that currently participate in the curriculum at Monash University. The survey asked whether radiographer commenting was regularly performed at their site.

**Table 1:** Brief description of pathologies and normal cases participants were examined on

Category	Types
Fractures	Displaced and undisplaced; comminuted; pathological; intra‐articular; supracondylar; Monteggia; Colles; healing; greenstick; Barton's; Salter‐Harris types; avulsion; multiple fractures; pelvic fractures; hip; suspected non‐accidental injury; tibial plateau; depressed calcaneal; Lisfranc fracture‐dislocation; stress; base of fifth metatarsal; occult
Dislocations/subluxations	Anterior and posterior; lunate; perilunate; scapho‐lunate disassociation; symphysis pubis diastasis
Soft‐tissue signs	Joint effusions; lipohaemarthrosis; elevated fat pads; surgical emphysema
Normal including normal variants	Apophysis; bipartite/multipartite patella; os trigonum; ossification centres
Common pathologies	Leg‐Calve‐Perthe's disease; slipped upper femoral epiphysis

### Statistical analysis

Statistical analyses were performed using the Statistical Package for the Social Sciences (SPSS) (IBM, version 23, Chicago, USA), with a *P*‐value of less than .05 being considered statistically significant. Each of the pre‐ and post‐ tests and retention test comprised 25 cases scored at a maximum of 50 marks. Paired‐samples *t*‐tests were carried out to compare image interpretation scores between the start of module 1 to the end of module 2, and between the end of module 2 and 6 months later. SCOR scores were achieved by employing Meta‐Disc tools.^17^

## Results

The participants’ demographic characteristics indicated a variety of experience levels ranging from 2 years to more than 10 years post‐qualification.

**Figure 1:**
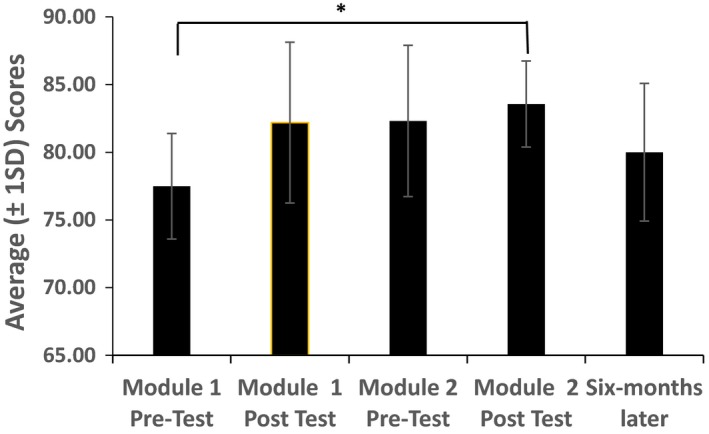
Mean (±SD) test scores of radiographers participating in an online image interpretation module (Total *N* = 8). *P = 0.022 (paired *t*‐test).

Figure 1 demonstrates the mean test scores (SD) of radiographers participating in two image interpretation modules. It demonstrates an improvement in mean score from pre‐test module 1 of 77.5 (±3.9) to the module 2 post‐test mean score of 83.6 (±3.2) (*P*=0.022).

Table 2 displays aggregated sensitivity (Sn), specificity (Sp) and accuracy (Acc) rates as well as likelihood ratios, negative predictive value (NPV) and positive predictive value (PPV) of radiographers’ participation in the two education modules. The PPV scored consistently above 95 for each of the module 1 and module 2 tests undertaken, confirming that participants were more accurate in identifying pathologies on test cases.

**Table 2:** Mean sensitivity (Sn), specificity (Sp), accuracy (Acc), negative (NPV) and positive predictive (PPV) values of radiographers participating in two online modules on image interpretation (Total *N* = 8)

	Sn	Sp	Acc	NPV	PPV
Pre‐test: module 1	82.28	75.29	81.68	79.24	97.23
Post‐test module 1	86.08	72.73	84.84	34.9	96.84
Pre‐test: module 2	85.66	81.53	84.97	53.22	95.86
Post‐test module 2	86.25	84.66	85.97	61.0	95.67
6 months later	83.51	68.33	81.34	40.85	94.05

Differences in test scores amongst radiographers participating in two online image interpretation modules, and 6 months later are shown in Table 3.

**Table 3:** Difference in test scores according to the timing of tests among radiographers participating in two online image interpretation modules (Total *N* = 8)

Paired *t*‐tests	*P*‐value*
Module 1 pre‐ versus post‐test	0.1
Module 2 pre‐ versus post‐test	0.563
Module 2 post‐test versus retention test	0.184
Module 1 pre‐test versus module 2 post‐test	0.022

* *P*‐value < 0.05

There was no statistically significant difference in scores from the start of module 1 to the end of module 1 (*P* =0.1). There was no statistically significant difference in scores from the start of module 2 to the end of module 2 (*P* =0.563). Similarly, scores between the end of module 2 and 6 months later were not statistically different (*P* =0.184). However, when comparing module 1 pre‐test scores to module 2 post‐test scores, scores improved significantly between the start of module 1 pre‐test to the end of module 2 post‐test (*P* =0.022).

**Figure 2:**
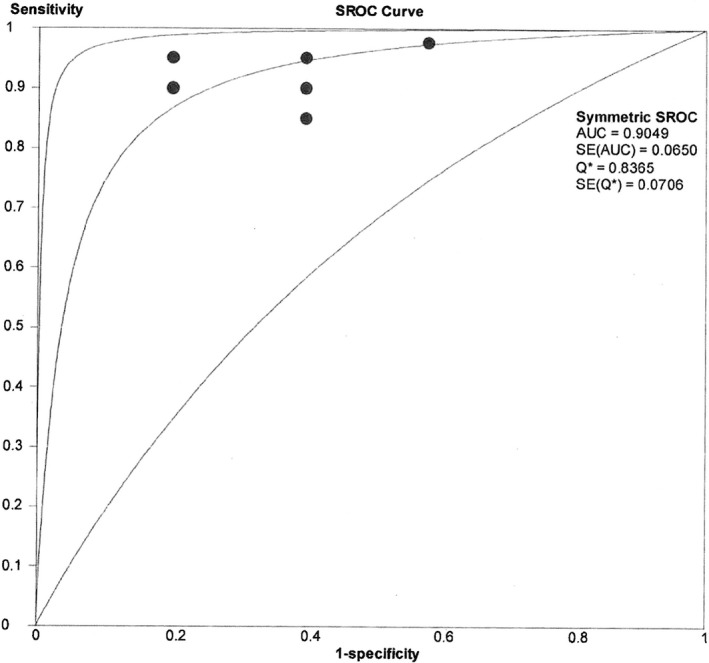
Module 2 post test pooled sensitivity and 1‐specificity summary receiver operating characteristics (SROC) curve.

When comparing the module 2 post‐test Sn and Sp for each participant as seen in Table 4, participant 2 has achieved the highest Sn (1.00) but also the lowest Sp (0.40). Apart from participant 5 (Sn = 0.85), all other participants achieved Sn scores of ≥0.90 which indicate higher accuracy in identifying pathologies when compared to the Sp scores. Figure 2 demonstrates a pooled area under the curve (AUC) of 0.90 achieved with analysis of the SROC curve. An AUC of 0.90 as a diagnostic performance indicates an almost perfect test with the AUC close to 1, as poor tests have AUCs close to 0.5. The Q* index achieved (0.84) which is the point where Sn and Sp are equal, was good (0.75–0.92)^18^ for this group of participants.

**Table 4:** Summary table of each participant's sensitivity and specificity achieved for module 2 post‐test

Participant	Sn (95% CI)	Sp (95% CI)
Participant 1	0.900 (0.683–0.988)	0.800 (0.284–0.995)
Participant 2	1.000 (0.832–1.000)	0.400 (0.053–0.853)
Participant 3	0.900 (0.683–0.988)	0.600 (0.147–0.947)
Participant 4	0.950 (0.751–0.999)	0.800 (0.284–0.995)
Participant 5	0.850 (0.621–0.968)	0.600 (0.147–0.947)
Participant 6	0.950 (0.751–0.999)	0.600 (0.147–0.947)
Participant 7	0.950 (0.751–0.999)	0.800 (0.284–0.995)
Participant 8	0.900 (0.683–0.988)	0.800 (0.284–0.995)

Of the 129 medical imaging sites, 52 (40%) responded to the straw poll survey. Of those, eight (15.4%) disclosed that radiographer commenting was routinely performed, whilst 25 (48.1%) might or might not perform radiographer commenting. Nineteen sites (35.5%) never performed radiographer commenting.

## Discussion

The education modules undertaken for the pilot project were in support of the MRPBA expectation that radiographers must be able to convey significant findings on acute plain imaging cases. In addition to identifying whether each case in our tests were normal or abnormal, participants had to provide a succinct comment on each of the cases. This approach provides an opportunity for participants to improve on their written commenting skills when interpreting radiographic images and conveying significant findings. The literature reveals a wide variation in normal to abnormal prevalence when compiling image test cases.^12^, ^19^, ^20^ In our study, we have used a ratio that is commonly encountered in clinical practice.^20^ The participants’ image interpretation scores improved across the course of the project. The module 1 pre‐test score demonstrated that 6 of the 8 participants achieved mean scores below 80% (75.8 (±3.9)), ranging from 71.9% to 78.9%. However, upon completion of the second module, all 8 participants achieved mean test scores of 83.6 (±3.18), ranging from 80.5% to 89.5%. The Sn rate (95.2%) achieved upon completion of the two modules was higher than the Sp rate (81.3%). This would indicate that participants were more confident in identifying abnormal cases in comparison to normal cases. Nevertheless, it also appears that participants lose some of their module‐based knowledge after 6 months. This may appear to be a disappointing result as the expectation would be that radiographers following training in image interpretation would improve on their commenting accuracy with further experience. However, the result is expected because on further investigation, Frankston Medical Imaging confirmed that they have not yet implemented radiographer commenting into their practice. This situation supports the long held belief that episodic practice does not assist with skill maintenance and development. It reinforces the need for continuing professional development to maintain skills acquired in the workplace. The informal straw poll of Victorian (VIC) Medical Imaging practices (*n* = 52), indicated that radiographer commenting across Victoria is only routinely performed in a minority of sites. A recent study also found that radiographer abnormality detection systems (RADS) are not extensively used in Queensland public hospitals with only 16% (*n* =4/25) of medical imaging directors reporting that RADS was in operation.^21^ However, evaluation of the impact of a pilot education programme on Queensland radiographers’ abnormality description of adult appendicular musculo‐skeletal trauma demonstrated that with appropriate education, almost all radiographers (*n *= 9/10) can match radiologists’ descriptions of appendicular musculo‐skeletal trauma.^22^ The obligation to pass on useful insights regarding image findings is now compulsory and Australian radiographers need to align their practice with the MRPBA expectations.^23^ Participants achieved significant improvements in commenting accuracy on appendicular skeleton plain images after the completion of the two modules. This demonstrates that focused radiographer training can support timely commenting of plain images in emergency settings. Informal feedback suggests that the training program increased the radiographers’ confidence. Whilst radiographer commenting cannot entirely replace radiologists’ reports, radiographers can and should provide timely support for cases with significant findings in the emergency department as routine practice and in particular when radiologists are not available.

## Limitations and Recommendations

This study only had a sample size of eight and we cannot generalise these findings to the Australian radiography profession. It is worthwhile noting that participant 2 with a Sn score of 100% and Sp score of 40% may have skewed the module 2 post‐test mean Sn and Sp scores due to the small sample size. In addition, participants were drawn from a single medical imaging site, which is not representative of medical imaging sites across the state of Victoria. It is recommended that a repeat of a similar study should include a larger sample size undertaken at multiple medical imaging sites representative of different clinical settings.

## Conclusion

The use of two online teaching modules improved radiographers’ ability to comment on plain images of the appendicular skeleton in the emergency setting with accuracy rates comparable to the radiologists’ reports. These findings suggest that the radiographers who completed the course should be able to identify significant findings and provide written comments with improved accuracy when operating in emergency settings. However, continuous application and ongoing professional development is essential in order to maintain and develop the skills acquired.

## Conflict of Interest

The authors declare no conflict of interest.
